# Sauti Za Wananchi “voice of the people”: patient satisfaction on the medical wards at a Kenyan Referral Hospital

**DOI:** 10.11604/pamj.2014.18.308.4466

**Published:** 2014-08-18

**Authors:** Geren Starr Stone, Tecla Sum Jerotich, Betsy Rono Cheriro, Robert Sitienei Kiptoo, Susie Joanne Crowe, Elijah Kipkorir Koros, Doreen Mutegi Muthoni, Paul Theodore Onalo

**Affiliations:** 1 Department of Medicine, Division of General Medicine, Center for Global Health and Disaster Response, Massachusetts General Hospital, Boston, Massachusetts; 2 Harvard Medical School, Boston, Massachusetts; 3 Department of Medicine, Indiana University School of Medicine, Indianapolis, Indiana; 4 School of Medicine, Department of Medicine, College of Health Sciences, Moi University, Eldoret, Kenya; 5 Moi Teaching and Referral Hospital, Eldoret, Kenya; 6 College of Pharmacy, Purdue University, West Lafayette, Indiana

**Keywords:** Patient satisfaction, global health, underserved population, sub-Saharan Africa

## Abstract

**Introduction:**

Patient satisfaction is one indicator of healthcare quality. Few studies have examined the inpatient experiences in resource-scarce environments in sub-Saharan Africa.

**Methods:**

To examine patient satisfaction on the public medical wards at a Kenyan referral hospital, we performed a cross-sectional survey focused on patients’ satisfaction with medical information and their relationship with staffing and hospital routine. Ratings of communication with providers, efforts to protect privacy, information about costs, food, and hospital environment were also elicited.

**Results:**

Overall, the average patient satisfaction rating was 64.7, nearly midway between “average” and “good” Higher rated satisfaction was associated with higher self-rated general health scores and self-rated health gains during the hospitalization (p = 0.023 and p = 0.001). Women who shared a hospital bed found privacy to be “below average” to “poor” Most men (72.7%) felt information about costs was insufficient. Patients rated food and environmental quality favorably while also frequently suggesting these areas could be improved.

**Conclusion:**

Overall, patients expressed satisfaction with the care provided. These ratings may reflect modest patients’ expectations as well as acceptable circumstances and performance. Women expressed concern about privacy while men expressed a desire for more information on costs. Inconsistencies were noted between patient ratings and free response answers.

## Introduction

Patient satisfaction is one indicator of healthcare outcomes and a measure of healthcare quality. Research suggests that satisfied patients are more likely to comply with prescribed treatments, provide information to healthcare providers, and continue using medical services [1, 2]. Few studies, however, have examined the experiences of inpatients in sub-Saharan Africa. Patient satisfaction is likely highly dependent on a number of factors including patient expectations, demographics, and psychosocial traits as well as healthcare worker traits and the hospital environment [3]. Moreover, patient satisfaction may be influenced by cultural background, self-interest, gratitude, and even the “Hawthorne effect” which postulates the additional attention implicit in satisfaction data-gathering leads to a more positive perception of services [3–5]. The objective of this study was to examine patient satisfaction on the public medical wards at a Kenyan referral hospital. In particular, the goal was to examine the level of patient satisfaction with respect to the quality of medical information provided and the patients’ relationships with healthcare providers and the daily hospital routine.

## Methods

### Study design and setting

This was a cross-sectional survey performed over 3 weeks in May 2013 via one-on-one patient interviews on the public medical wards of Moi Teaching and Referral Hospital (MTRH) in Eldoret, Kenya. MTRH is an approximately 750-bed national referral hospital for western Kenya. Divided into men's and women's wards, the medical wards together admit approximately 400 patients monthly. These wards are largely populated by those in the lowest socioeconomic strata as those with means largely choose private wards or hospitals [6]. On these wards, patients often reside two per bed, lying head-to-foot. Food is provided by the hospital with supplemental food from outside prohibited. Nursing staff-to-patient ratios are unfavorable with each nurse attending to approximately 15 patients. Medical staffing is provided by teams, including medical officer interns, post-graduate registrars, and attending consultants. Medications and supplies are subject to periodic stock outs. In the midst of this environment on the public medical wards, this study sought to examine the patients? perspectives and their satisfaction with respect to their care. The study was approved by the local ethics committee at Moi University and the Institutional Review Board at Indiana University. Moreover, permission was granted from the MTRH administration for the study.

### Population

Using “Quick Random Number Generator” by CWE software for Android operating system, each work day during the study period -Monday to Friday- bed numbers were randomly selected for the men's and women's wards separately along with a window/aisle designation. The interviewers visited each bed chosen in order until 3 patient interviews had been completed on each ward daily. A total of 90 randomly selected patients (45 men and 45 women) were interviewed.

Patients were excluded if they were unable or unwilling to consent or participate in the 10-15 minute interview (ie. critically ill, confused, unable to communicate, language barrier) or if they were prisoners, younger than 18 years old, or discharged yet still on the wards awaiting financial release. If the patient was not present in his/her bed, the research team tried to find the patient before moving on to the next randomly selected bed. Patients were only eligible to respond to the survey on a single occasion. All interviewers performing the surveys were fluent in Kiswahili, English, and the tribal language most common locally.

### Data collection

Interviews were structured around a modified 16-question Echelle de Qualité des Soins en Hospitalisation (EHQ-S) survey with additional questions focused on the patient knowledge of the healthcare providers’ names, perspective on communication with the providers, understanding of hospital costs, and perspective on the hospital's food, cleanliness, and efforts to ensure privacy [7]. The EQS-H is one well-known and validated scale used to assess in patient satisfaction in various settings. Through validation studies in other settings, it has been reduced to 16 items covering 2 domains of patient satisfaction: quality of medical information and relationship with staff and daily hospital routine [7]. Each domain consists of 8 items with each rated on a five-point Likert scale. The final 3 questions of the medical information subset focus specifically on discharge information as the EHQ-S is generally used at the time of discharge or afterwards [8, 9].

In the survey, demographic information, including age, gender, educational level, marital status, insurance status, occupation, and urban/rural residence, was also collected. A local Kiswahili expert translated all questions into Kiswahili, and visual analogue scales were added as tools of reference to aid patient understanding of the Likert scale. For patients preparing for imminent discharge within 24 hours of survey, all questions within the EHQ-S survey were used. Meanwhile, the three questions on discharge information and planning were withheld from the other patients. Interviews were conducted by non-clinical personnel in a manner protecting the privacy of patients with verbal consent obtained before beginning the survey.

### Statistical analysis

Responses on the EQH-S were analyzed numerically with each choice assigned a point value. For the 5-point Likert scale, a rating of “very poor”: was assigned 0 points; “poor”: 1 point; “average”: 2 points; “good”: 3 points and “very good”: 4 points. For each patient, an overall patient satisfaction score out of 100 was then calculated with total points divided by number of possible points with 0 equivalent to “very poor“; 25 to “poor”; 50 to “average”; 75 to “good” and 100 to “very good”.

Univariate analyses were undertaken using a 2-sample t-tests and ANOVA tests. Differences in overall patient satisfaction were compared between subgroups based on demographic characteristics, length of stay, self-rated health status, self-rated improvement during hospitalization, and a history of having shared a bed with another patient while hospitalized. The overall scores then also underwent multivariate linear regression analysis with respect to these variables. Analysis of the patient satisfaction scores was undertaken within subscale indices of the medical information and relationship to staffing. A separate analysis focused specifically on the quality of discharge information. For each analysis, an overall score, standard deviation, and range was generated. The questions’ remainder were analyzed separately from the modified EQH-S questions using chi-square test along with 5-point and 3-point Likert scales similarly scored as above. All analyses were conducted with SPSS software (Version 20; SPSS Inc).

## Results

During the study period, 45 men and 45 women completed the survey with 195 beds randomly chosen excluded: 29 beds had patients who had previously participated in the study, 98 beds had patients either unable or unwilling to consent, 39 beds were either unoccupied or the interviewer was unable to locate patient, 11 beds were occupied by patients either <18 years old or prisoners, 14 beds had patients discharged but awaiting financial release, and 4 beds were excluded for unclear reasons. One survey was misplaced during the study with an additional randomly selected bed on a subsequent day chosen.

### Study population

Compared to the women, the men surveyed were older, and the men were also more likely to be married, employed outside the home, and to be insured (Table 1). Not reaching statistical significance, the men also were also more likely to have completed some secondary school and had a shorter length of stay at the time of being surveyed. Women though generally rated their health status slightly higher. More than two-thirds of both lived in rural areas. Furthermore, similar percentages of men and women interviewed shared a bed during their hospitalizations.

**Table 1:** Study population

	Men	Women	Difference	Entire population
**N**	45	45	—	90
**Age**	44.9 (18-87, SD 16.6)	37.1 (18-85, SD 15.7)	+7.7 (p = 0.03)	41.1 (18-87, SD 16.5)
**Married**	33 (73.3%)	20 (44.4%)	+28.9% (p< 0.005)	53 (58.9%)
2° Education*	27 (60.0%)	20 (44.4%)	+15.6% (p = 0.17)	47 (52.2%)
**Employed**	36 (80.0%)	15 (33.3%)	+46.7% (p < 0.005)	51 (56.7%)
**Uninsured**	26 (57.8%)†	32 (71.1%)	-13.3% (p = 0.01)	58 (64.4%)
**Rural**	31 (68.9%)	35 (77.8%)‡	-8.9% (p = 0.67)	66 (73.3%)
**Length of Stay**	6.2 (1-34, SD 7.6)	10.1 (2-56, SD 11.1)	-3.8 (p = 0.06)	8.2 (1-56, SD 9.7)
**Shared Bed**	30 (66.7%)	31 (68.9%)	-2.2% (p = 0.82)	31 (67.8%)
**Avg% Days Shared Bed**§	82% (SD 25.6)	93.85% (SD 23.4)	-11.8% (p = 0.09)	87.9% (SD 26.9)
**Health Status**	5.8 (3-10, SD 1.9)	6.5 (3-10, SD 1.7)	-0.7 (p = 0.054)	6.2 (3-10, SD 1.8)

* At least partial secondary education completed.

† Missing insurance information for 7 female patients.

‡ Missing residential information for 3 male patients.

§ Only included those patients that shared beds in this calculation

### Overall patient satisfaction ratings

**Figure 1:**
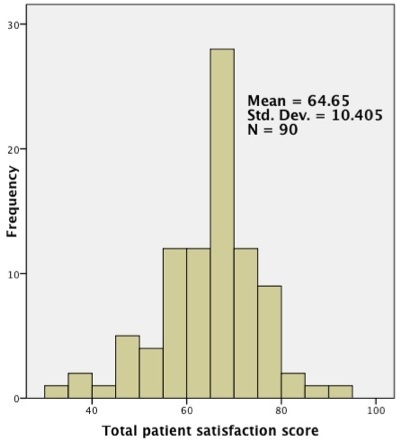
Total patient satisfaction: the distribution of total patient satisfaction ratings

The average patient's overall satisfaction rating for this population was approximately midway between “average” and “good” (Figure 1). The mean overall rating was 64.7 (range 34.6-94.2) as derived on a 100-point scale. No significant differences in total satisfaction ratings were seen based on gender, age, marital status, education level, employment status, insurance status, place of residence, length of stay, or whether a patient shared a bed during the hospitalization.

ANOVA tests revealed a statistically significant difference in total satisfaction ratings based on general self-rated health status ratings (p = 0.02) and self-rated improvement during hospital stays (p = 0.001). Increased satisfaction was associated with higher self-rated scores for general health status and improvement. In standard multivariate linear regression examining the association between all the variables examined above and overall patient satisfaction, the model did reach statistical significance (p = 0.018); however, no individual factor reached statistical significance.

### patient satisfaction subscales

Similar scores to the overall satisfaction score were seen on the subscale analyses for quality of medical information (mean 61.7) and the patients’ relationships with staff and daily hospital routine (mean 66.5). When discharge information was examined as its own subscale, it had a similar score with a mean of 66.7. However, only 17 patients were expecting discharge in the subsequent 24 hours and were eligible for these questions.

### Communication with healthcare providers

Ten (11.1%) patients -including only 1 male- surveyed were able to name their nurse. One nurse was named by 4 patients and another named by 3 patients. Nineteen (21.1%) patients surveyed were able to name one of their doctors. This represented 8 physicians’ names, including 1 physician named by 10 different patients. However, despite so few being able to name their healthcare providers, still 69 (76.7%) and 66 (73.3%) patients, respectively, rated the amount of time spent communicating with their nurse and doctor as “optimal“. No significant differences were seen based on gender.

### Privacy

**Figure 2:**
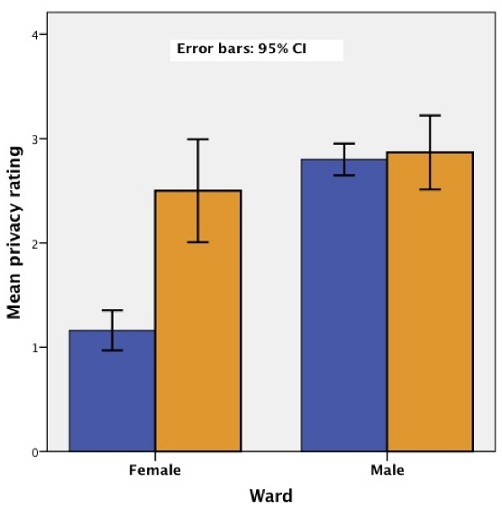
Hospital efforts to protect patients’ privacy: the mean rating for women and men categorized by whether they shared a hospital bed is shown above

The average rating for efforts to ensure privacy on the wards was 2.20 on the 5-point scale. With a mean score of 1.58, women's ratings were significantly lower than the men's mean of 2.82 (p < 0.005). The women who shared a bed during their hospitalizations rated privacy efforts significantly lower compared to those that did not (1.16 vs 2.50, p < 0.005). Of the 31 women who shared a bed, 26 (83.9%) rated efforts to ensure privacy as “poor” or “very poor”. The ratings of women who did not share a bed were not significantly different from the men including when compared to those who did and did not share a bed (Figure 2). Meanwhile, when asked in an open-ended fashion about priorities for hospital improvement, 11 (12.2%) patients, including 10 women, mentioned improving privacy.

**Figure 3:**
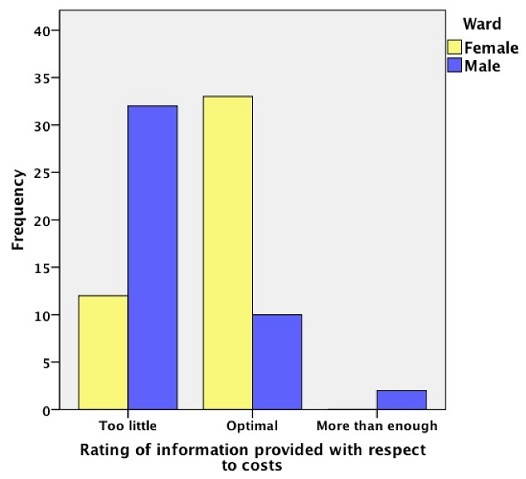
Information about hospital costs: the distribution of ratings for women and men

***Costs:*** Forty-four (48.9%) patients rated the information provided about hospital costs as “too little”. Only 12 (26.7%) women rated the information as “too little” compared to 32 (72.7%) men (p < 0.005) (Figure 3).

***Food and environment:*** The majority of respondents rated food services as “average” to “very good” (n = 76, 84.4%). Similarly, 81 (90%) patients rated the wards’ cleanliness as “good” to “very good”. Yet, 40 (44%) of the patients interviewed on open-ended questioning mentioned food, space, or the hospital's physical environment as priority areas for improvement.

***Other areas:*** Other areas for improvement mentioned by patients to open-ended questioning included increased space and beds (n = 19).

## Discussion

Patient satisfaction is one aspect in assessing the quality of care. Measurements of patient satisfaction allow for description of healthcare services from the patients’ perspectives, offer insight into problem areas and possible solutions, and aid in the evaluation of quality of care [3]. However, few studies have examined the experiences of inpatients in sub-Saharan Africa and other resource-scare settings.

Our analysis using the EQS-H and focusing on adult inpatients on the public medical wards at a Kenyan national referral hospital demonstrated an overall general satisfaction expressed by the patients with a mean satisfaction score of 64.7. This rating correlated to nearly midway between “average” and “good“ On analyses, the overall score was found only to be related to self-rated general health status and self-rated clinical improvement during hospitalization. This overall measure was derived from two indices of quality of medical information and the patients’ relationship with staff and daily hospital routine, both of which showed similar mean subscale scores of 61.7 and 66.5.

Generally, these findings match with a satisfaction rating of 66.8% found by Iloh and colleagues at a tertiary hospital in southeastern Nigeria. They found respondents expressed satisfaction with patient-provider relationship (81.5%), patient-provider communication (79.9%), accessibility (74.2%), and hospital environment (68.2%) [10]. A similar study performed at a referral hospital in Dar Es Salaam, Tanzania found most patients to be satisfied with the services and care provided. In that study, 91.7% of patients on the medical wards rated their overall care by physicians as “very good” or “excellent” without a single rating of “fair” or “poor”. Ratings for nursing care were slightly lower with 78.8% rating “very good” or “excellent” and 4.5% “fair” or “poor”. In general, patients felt services were good quality and superior to lower-level health facilities; though still some expressed dissatisfaction with long wait times, high costs, poor levels of hygiene on wards, and negative staff attitudes [11]. In contrast, Ofuvwe and Ofili found only 45.1% of patients on the medical wards at another tertiary health facility in Nigeria reported receiving adequate medical information during their hospitalization [12]. Cultural context, survey tool, setting of survey within hospital (ie. outpatient, surgical wards), and timing of survey (eg. post-discharge) may all help to explain the different findings among these studies and our results.

In our study, while only 11.1% and 21.1% of patients could name their nurse or physician, patients still expressed overall satisfaction with communication with their healthcare providers. Interestingly, only 1 male patient could name his nurse, and more than double the number of patients could name their physician than their nurse. However, 1 physician represented over half of the physicians named.

Overall, women who shared a bed with another patient noted privacy to be a problem compared to men and other women. Men, on the other hand, more often rated the amount of information about hospital costs as “too little“. The populations of men and women chosen randomly differed on a number of measures. The men surveyed were older, more likely to be married, more likely to be employed outside the home, and more likely to be insured. This likely reflects underlying differences in the populations admitted to the two wards at this hospital.

The majority rated food services (84.4%) and wards’ cleanliness (98.9%) as “average” or better. Yet, 44% of patients on open-ended questioning mentioned food, space, or the hospital's physical environment as priority areas for improvement. This discrepancy illustrates how responses may vary in this context with question format and framing, and it encourages caution in drawing strong conclusions from ratings alone.

Even in high-income settings, there is concern about the interpretation of patient satisfaction with questions to its correlation with health outcomes [13]. Generally, patient satisfaction surveys consistently report high levels of patient satisfaction [14, 15]. Moreover, as researchers have demonstrated, patient-described experiences do not always correlate with their evaluations of the very services that produced those experiences. As Williams et al wrote, “high satisfaction ratings do not necessarily mean that patients have had good experience in relation to the service”. Rather it may reflect a general sense that healthcare providers are “doing the best they can” [16]. Moreover, there may be a tendency due to gratitude, fear, or culture to withhold critical and negative comments [4, 14]. Additionally, patient satisfaction measurements have been found to vary with patient socio-demographic variables [17, 18]. They also have been found to vary with respect to length of stay, previous admissions, timing of response to questionnaire, clinical outcome, and current health status [17, 19].

Our study had a number of limitations. The population surveyed was limited to 45 male and 45 female patients on the medical wards. We did not survey patients on any of the other wards or outpatient areas. Secondly, the survey used -the EQS-H- has been validated in other settings but never before in sub-Saharan Africa. Moreover, variables such as number of past admissions, reasons for current admission, or income level were not examined in our present study. In general, this population on the public wards represents low-income levels with emergency/unplanned admissions. However, these variables, especially experiences during past admissions or at other health centers, very well could affect patients’ expectations and perceptions of their care. Additionally, subtracting the unoccupied beds, patients selected multiple times, and those post-discharge awaiting financial release, still 39.5% of patients randomly selected were excluded. The majority (86.7%) of these were due to inability or unwillingness to consent which included confusion, language barriers, and clinical conditions prohibiting participation. Finally, we interviewed patients in the midst of their hospitalization. Only 17 (18.9%) of the patients interviewed expected to be discharged in the subsequent 24 hours. This severely limited our ability to assess quality of discharge information and gather a summary perspective of entire hospitalizations. Furthermore, while we attempted to ensure privacy during interviews and assured confidentiality, patients may have been reticent to provide critical or negative remarks in the midst of receiving care on the wards.

Yet, we believe our study had a number of strengths, including utilizing a validated, short, and easy-to-understand survey along with the addition of visual analogue scales. Though only including 90 patients admitted over a period of 3 weeks, this does represent a significant portion of total admissions to the public medical wards for the month. Furthermore, using personal interviews, we had a very low non-response rate to questions.

## Conclusion

Patients on the public medical wards expressed a general consensus that the care they were receiving was above average. They did so in a setting where resources are limited, patient-to-staff ratios are often quite high, and space requirements lead to patients often sharing beds. This patient consensus can be seen as a validation of the care provision currently; however, we also see it at as a sobering reflection on the current level of expectations for care provision by these patients. As stated above, their evaluations may reflect a general consensus that everyone is “doing the best he can” in a difficult system [16]. Likewise, positively reported patient satisfaction should not be used to cover over hidden problems of care provision such as lack of resources, non-adherence to guidelines, or other areas for improvement in care [20]. In the end, while overall expressing general satisfaction, our patients also noted areas for improvement, including privacy and information with respect to hospital costs. Areas for future research and exploration include the means to best elicit and evaluate patient satisfaction in this setting and the variables that shape their expectations and ratings.
